# *CYP3A4* and *CYP11A1* variants are risk factors for ischemic stroke: a case control study

**DOI:** 10.1186/s12883-020-1628-4

**Published:** 2020-03-04

**Authors:** Ning Gao, Hong Tang, Ling Gao, Guolong Tu, Han Luo, Ying Xia

**Affiliations:** Department of Neurosurgery, Affiliated Haikou Hospital of Xiangya Medical College of Central South University, Haikou People’s Hospital, #43, People’s Avenue, Haidian Island, Haikou, 570208 Hainan China

**Keywords:** Ischemic stroke, *CYP3A4*, *CYP11A1*, Polymorphism, Susceptibility

## Abstract

**Background:**

This study aimed to investigate the roles of *CYP3A4* and *CYP11A1* variants in ischemic stroke (IS) susceptibility among the Han Chinese population.

**Methods:**

Four hundred seventy-seven patients with IS and 493 healthy controls were enrolled. Seven single-nucleotide polymorphisms (SNPs) of *CYP3A4* and *CYP11A1* were genotyped by Agena MassARRAY. Odds ratio (OR) and 95% confidence intervals (CI) were calculated by logistic regression adjusted for age and gender.

**Results:**

We found that *CYP3A4* rs3735451 (OR = 0.81, *p* = 0.039) and rs4646440 (OR = 0.72, *p* = 0.021) polymorphisms decreased the risk of IS. *CYP3A4* rs4646440 (OR = 0.74, *p* = 0.038) and *CYP11A1* rs12912592 (OR = 1.58, *p* = 0.034) polymorphisms were correlated with IS risk in males. *CYP3A4* rs3735451 (OR = 0.63, *p* = 0.031) and rs4646440 (OR = 0.57, *p* = 0.012) possibly weaken the IS susceptibility at age > 61 years. Besides, *CYP3A4* rs4646437 (OR = 0.59, *p* = 0.029), *CYP11A1* rs12912592 (OR = 1.84, *p* = 0.017) and rs28681535 (OR = 0.66, *p* = 0.038) were associated with IS risk at age ≤ 61 years. *CYP11A1* rs28681535 TT genotype was higher high-density lipoprotein cholesterol level than the GT and GG genotype (*p* = 0.027).

**Conclusions:**

Our findings indicated that rs3735451, rs4646440, rs4646437 in *CYP3A4* and rs28681535 in *CYP11A1* might be protective factors for IS, while *CYP11A1* rs12912592 polymorphism be a risk factor for IS in Chinese Han population.

## Background

Stroke, a common multifactor neurological disease, is a common cause of death and severe disability in adults worldwide. The incidence of stroke is estimated to be more than 2 million people and more than 1 million people die from stroke-related causes every year in the Chinese population [1]. There are huge economic and social burdens because of stroke in China, which remains particularly high in the northern and central regions [2]. Ischemic stroke (IS) is the most common type of stroke accounting for 80–85% of all stroke cases [3]. According to epidemiologic studies, the incidence of IS in China is significantly higher than in developed countries [4]. The pathophysiological causes of IS are unclear, but the widely accepted concept is that IS is caused by the interaction between genetic and environmental factors [5]. To date, many studies have identified that gene polymorphisms modulate the pathophysiological processes of IS and confer a small to moderate risk [6–8].

Cytochrome P450s (CYPs) is a group of complexes and structurally related enzymes with diverse metabolic and biosynthetic activities. CYP epoxygenases is metabolizing arachidonic acid (AA) to biologically active epoxyeicosatrienoic acids (EETs), which exert vascular relaxation effects and have diverse protective roles in the cardiovascular system [9]. Previous studies have shown that plasma CYP metabolite levels, including EETs are associated with IS [10, 11]. *CYP3A4* gene, located on chromosome 7q21.1, is a member of the *CYP3A* gene family, which participates in metabolizing arachidonic acid (AA) into epoxyeicosatrienoic acids (EETs) [12]. *CYP11A1* gene is located on chromosome 15q23-q24, and is involved in the metabolism of cholesterol and vitamin D, which associated with cardiovascular diseases [13, 14]. Consequently, studies concerning the possible association of *CYP3A4* and *CYP11A1* gene with IS may be particularly interesting for their potential biological significance.

However, few reports concerning the role of *CYP3A4* and *CYP11A1* polymorphisms on IS risk have been published yet. Therefore, we carried out a case-control study to explore whether polymorphisms in *CYP3A4* and *CYP11A1* contribute to the risk of IS in a Chinese Han population.

## Methods

### Study participants

A cohort of 477 IS patients and 495 control subjects were enrolled from Haikou People’s Hospital and the Affliated Hospital of Yan′an University in this study. All recruited subjects were unrelated ethnic Han Chinese. All the patients were identified as having newly diagnosed IS by at least two independent neurologists, according to the clinical signs and symptoms. All patients underwent brain computed tomography (CT) scans and/or magnetic resonance imaging (MRI) as well as standardized clinical hematology, biochemistry and immunology examinations. Patients with a history of hematologic, coronary artery diseases, autoimmune diseases, systemic inflammatory diseases, blood diseases, or malignant tumors were excluded. The healthy individuals without the history of stroke, normal neurological examination results, and free from cardiovascular and cerebrovascular diseases, and immunological diseases, who received a physical examination in the same hospital, were recruited as controls. Demographic characteristics, clinical information and medications were collected with standardized questionnaires. The following clinical data were collected: age, gender, total protein, serum uric acid, blood glucose, total bilirubin, total cholesterol, triglyceride, low-density lipoprotein and high-density lipoprotein. This study protocol was approved by the Ethics Committee of Haikou People’s Hospital and was conducted according to the guidelines on the Declaration of Helsinki. Informed consent was obtained from all participants.

### Sample collection and SNP genotyping

Blood samples were obtained from the peripheral veins and were stored in EDTA-coated tubes at -80C until further analysis. Genomic DNA was isolated from peripheral blood samples using the GoldMag DNA Purification Kit (GoldMag Co. Ltd., Xi’an City, China) according to the manufacturer’s instructions. The DNA concentration and purity was determined using NanoDrop 2000 (Thermo Scientifc, Waltham, MA, USA). Four *CYP3A4* SNPs (rs3735451, rs4646440, rs35564277 and rs4646437) and three *CYP11A1* SNPs (rs1484215, rs12912592 and rs28681535) were selected based on the NCBI SNP database and minor allele frequencies (MAFs) > 5% in the 1000 Genomes Project data (http://www.internationalgenome.org/). In order to uncover the functional effects of *CYP3A4* and *CYP11A1* polymorphisms, online software for HaploReg v4.1 (https://pubs.broadinstitute.org/mammals/haploreg/haploreg.php) was used.

The MassARRAY platform is based on MALDI-TOF (matrix-assisted laser desorption/ionization—time of flight) mass spectrometry in a high-throughput and cost-effective manner [15, 16]. The advantage of MassARRAY platform is 1) multiplex PCR assays for up to 40 SNPs simultaneously; 2) relatively forgiving in terms of required DNA quality and quantity; 3) The primers design and the data management were implemented using Agena Design 3.0 Software and Agena Typer 4.0 software, respectively. Therefore, in our study, SNPs genotyping were performed using Agena MassARRAY system (Agena, San Diego, CA, U.S.A.) as previously described, and conducted by laboratory technicians blinded to the case–control status. The primers for PCR amplification and single base extension were present in Additional file 1: Table S1. Approximately 10% of samples were randomly selected to repeat genotyping for quality control, and a 100% concordant was achieved.

### Data analysis

Statistical analyses were performed using SPSS version 18.0 (SPSS Inc., Chicago, IL, USA) and PLINK software. Demographic data of patients and controls were compared using student’s t-test and chi-square test. Hardy–Weinberg equilibrium (HWE) was examined via a goodness-of-fit χ^2^ test to compare the observed genotype frequencies and the expected frequencies among the control subjects. The genotype and allele frequencies of the controls and IS patients were compared using the χ^2^ test or Fisher’s exact test. The correlation between *CYP3A4* and *CYP11A1* polymorphisms and IS susceptibility was estimated by odds ratios (ORs) and 95% confidence intervals (CIs) using logistic regression analysis with adjustment for age and sex by PLINK software. Multiple inheritance models (genotype, dominant, recessive and log-additive) were estimated. Further, we calculated stratification factors using age (≤ 61 and > 61 years) and gender (male and female) to adjust for possible cofounders. Pairwise linkage disequilibrium (LD) between the selected SNPs was measured by the LD coefficient D’ using the Haploview software (version 4.2), and haplotype analyses were performed by logistic regression analysis using the PLINK software. Finally, the association between the genotypes of *CYP3A4* and *CYP11A1* polymorphisms and clinical parameters was tested by covariance analysis (ANCOVA). A two-tailed *p*-value < 0.05 was considered as significant.

## Results

In total, 477 IS patients (316 males and 161 females) and 493 control subjects (325 males and 168 females) were recruited. There were no significant differences between patients and controls in terms of gender (*p* = 0.898). The mean age was 64.13 ± 10.82 years for the patients with IS and 60.05 ± 6.56 years for the control subjects. Significant differences were also found in age distribution (*p* < 0.001), suggesting that age may have an effect on the etiology of IS. The total protein, serum uric acid, blood glucose, bilirubin, triglyceride, hemoglobin, cholesterol and low-density lipoprotein levels in the IS patients were significantly different from those noted in the healthy control subjects. The clinical characteristics of the patients were described in Table 1.

**Table 1:** Characteristics of patients with ischemic stroke and controls

Characteristics	Cases (*n* = 477)	Controls (*n* = 493)	*p*
Age, year (mean ± SD)	64.13 ± 10.82	60.05 ± 6.56	< 0.001
Gender (M/F)	316/161	325/168	0.898
TP (g/L, mean ± SD)	65.57 ± 5.80	70.88 ± 5.61	< 0.001
Serum uric acid (μmol/L, mean ± SD)	284.53 ± 94.37	330 ± 80.27	< 0.001
Blood glucose (mmol/L, mean ± SD)	6.33 ± 2.24	5.83 ± 1.44	0.001
TB (μmol/L, mean ± SD)	13.63 ± 6.51	17.00 ± 5.94	< 0.001
TG (mmol/L, mean ± SD)	1.59 ± 1.05	4.50 ± 0.92	< 0.001
Hemoglobin (g/L, mean ± SD)	136.87 ± 22.77	147.76 ± 14.31	< 0.001
TC (mmol/L, mean ± SD)	3.89 ± 1.03	1.79 ± 1.16	< 0.001
HDL-C (mmol/L, mean ± SD)	1.09 ± 0.26	1.09 ± 0.23	0.871
LDL-C (mmol/L, mean ± SD)	1.81 ± 0.58	2.56 ± 0.71	< 0.001

*SD* standard deviation, *TP* total protein, *TB* total bilirubin, *TG* triglyceride, *TC* total cholesterol, *HDL-C* high-density lipoprotein cholesterol, *LDL-C* lowdensity lipoprotein cholesterol

Seven SNPs in *CYP3A4* and *CYP11A1* were successfully genotyped, and the average variant call rate was 99.6%. Detailed information and potential function of candidate SNPs were listed in Table 2. These intronic SNPs were associated with the regulation of promoter and/or enhancer histones, changed motifs, and selected eQTL hits, suggesting they might exert biology functions in silico. MAF of all SNPs was higher than 5% of the study population. All SNPs were in HWE among the controls (*p* > 0.05).

**Table 2:** The information about the candidate SNPs in *CYP3A4* and *CYP11A1*

Gene	SNP ID	Chr: Position	Alleles (minor/major)	Frequency (MAF)		HaploReg
				Case	Control	
CYP3A4	rs3735451	7:99758352	C/T	0.26	0.30	Motifs Changed, Selected eQTL hits
CYP3A4	rs4646440	7:99763247	A/G	0.19	0.23	Promoter histone marks, Enhancer histone marks, DNAse, Proteins bound, Motifs changed, Selected eQTL hits
CYP3A4	rs35564277	7:99764813	C/T	0.06	0.07	Motifs Changed
CYP3A4	rs4646437	7:99767460	A/G	0.11	0.13	Promoter histone marks, Enhancer histone marks, Motifs changed, Selected eQTL hits
CYP11A1	rs1484215	15:74347768	T/C	0.18	0.18	Enhancer histone marks, Motifs changed, Selected eQTL hits
CYP11A1	rs12912592	15:74363369	T/G	0.10	0.08	Enhancer histone marks, Motifs changed, Selected eQTL hits
CYP11A1	rs28681535	15:74367268	T/G	0.43	0.45	Promoter histone marks, Enhancer histone marks, DNAse, Motifs changed

*MAF* minor allele frequency, *eQTL* expression quantitative trait loci

The allele and genotype frequency distributions of the SNPs and their association with IS susceptibility were shown in Table 3 and Additional file 1: Table S2. *CYP3A4* SNPs rs3735451 and rs4646440 were associated with reduced susceptibility of IS (Table 3). We found that individuals carrying rs3735451-C allele had a decreased risk of IS in allele model (OR = 0.81, 95% CI: 0.66–0.98, *p* = 0.039), genotype model (OR = 0.74, 95% CI: 0.57–0.97, *p* = 0.029), dominant model (OR = 0.73, 95% CI: 0.56–0.95, *p* = 0.018) and additive model (rs3735451 OR = 0.78, 95% CI: 0.63–0.96, *p* = 0.019), respectively. With rs4646440 GG genotype as reference, the presence of the GA genotype was associated with a significantly decreased risk of IS after adjustment for age and gender (GA vs. GG, OR = 0.72, 95% CI: 0.55–0.95, *p* = 0.021; GA-AA vs. GG, OR = 0.72, 95% CI: 0.55–0.94, *p* = 0.017, Table 3). Furthermore, rs4646440 polymorphism also might reduce the susceptibility to IS under additive model (OR = 0.77, 95% CI: 0.61–0.97, *p* = 0.024). Nevertheless, other polymorphisms in *CYP3A4* and *CYP11A1* did not relate to IS susceptibility (Additional file 1: Table S2).

**Table 3:** Relationships between *CYP3A4* and *CYP11A1* polymorphism and ischemic stroke risk

Gene SNP ID	Model	Genotype	Case	Control	Adjusted by age and gender	
					OR (95%CI)	*p*
CYP3A4 rs3735451	Allele	T	705	686	1.00	**0.039**
		C	249	300	**0.81 (0.66–0.98)**	
	Genotype	TT	256	228	1.00	
		CT	193	230	**0.74 (0.57–0.97)**	**0.029**
		CC	28	35	0.66 (0.38–1.14)	0.135
	Dominant	TT	256	228	1.00	**0.018**
		CT-CC	221	265	**0.73 (0.56–0.95)**	
	Recessive	TT-CT	449	458	1.00	0.308
		CC	28	35	0.76 (0.45–1.29)	
	Log-additive	–	–	–	**0.78 (0.63–0.96)**	**0.019**
CYP3A4 rs4646440	Allele	G	768	754	1.00	**0.046**
		A	186	228	0.80 (0.64–1.00)	
	Genotype	GG	307	282	1.00	
		GA	154	190	**0.72 (0.55–0.95)**	**0.021**
		AA	16	19	0.72 (0.36–1.45)	0.362
	Dominant	GG	307	282	1.00	**0.017**
		GA-AA	170	209	**0.72 (0.55–0.94)**	
	Recessive	GG-GA	461	472	1.00	0.560
		AA	16	19	0.81 (0.41–1.62)	
	Log-additive	–	–	–	**0.77 (0.61–0.97)**	**0.024**

*SNP* single nucleotide polymorphism, *OR* odds ratio, *95% CI* 95% confidence interval

*p* values were calculated by logistic regression analysis with adjustments for age and gender

*p* < 0.05 means the data is statistically significant

Bold indicates that the values have statistical significance

We further analyzed whether the genotypic effects on IS risk were dependent on gender (Table 4). We found that *CYP3A4* rs4646440 was associated with a decreased risk under the additive model (OR = 0.74, 95% CI: 0.56–0.98, *p* = 0.038), and showed a marginal *p* value in allele model (OR = 0.76, 95% CI: 0.58–1.00, *p* = 0.050) among males, which indicated insufficient evidence for claiming an association. *CYP11A1* rs12912592 polymorphism also showed significant risk-increasing effects in the heterozygote model (OR = 1.58, 95% CI: 1.04–2.42, *p* = 0.034), and dominant model (OR = 1.56, 95% CI: 1.02–2.37, *p* = 0.039).

**Table 4:** Relationships between *CYP3A54* and *CYP11A1* polymorphism and ischemic stroke risk according to the stratification by gender

SNP ID	Model	Genotype	Male				Female			
			Case	Control	OR (95%CI)	*p*	Case	Control	OR (95%CI)	*p*
CYP3A4 rs4646440	Allele	G	509	492	1.00	0.050	259	262	1.00	0.563
		A	123	156	0.76 (0.58–1.00)		63	72	0.89 (0.61–1.29)	
	Genotype	GG	202	183	1.00	0.117	105	99	1.00	0.226
		GA	105	126	0.74 (0.53–1.05)	0.089	49	64	0.69 (0.43–1.11)	0.123
		AA	9	15	0.53 (0.22–1.27)	0.156	7	4	1.44 (0.40–5.15)	0.576
	Dominant	GG	202	183	1.00	0.052	105	99	1.00	0.188
		GA-AA	114	141	0.72 (0.52–1.00)		56	68	0.74 (0.47–1.16)	
	Recessive	GG-GA	307	309	1.00	0.237	154	163	1.00	0.438
		AA	9	15	0.60 (0.25–1.41)		7	4	1.65 (0.47–5.81)	
	Log-additive	–	–	–	**0.74 (0.56–0.98)**	**0.038**	–	–	0.83 (0.56–1.24)	0.371
CYP11A1 rs12912592	Allele	G	565	602	1.00	0.081	297	301	1.00	0.672
		T	67	50	1.43 (0.97–2.10)		25	29	0.87 (0.50–1.53)	
	Genotype	GG	250	277	1.00		138	136	1.00	
		GT	65	48	**1.58 (1.04–2.42)**	**0.034**	21	29	0.64 (0.34–1.19)	0.161
		TT	1	1	0.63 (0.04–10.19)	0.743	2	0	/	/
	Dominant	GG	250	277	1.00	**0.039**	138	136	1.00	0.224
		GT-TT	66	49	**1.56 (1.02–2.37)**		23	29	0.70 (0.38–1.28)	
	Recessive	GG-GT	315	325	1.00	0.704	159	165	1.00	/
		TT	1	1	0.58 (0.04–9.45)		2	0	/	
	Log-additive	–	–	–	1.51 (1.00–2.28)	0.051	–	–	0.78 (0.44–1.38)	0.393

*SNP* single nucleotide polymorphism, *OR* odds ratio, *95% CI* 95% confidence interval

*p* values were calculated by logistic regression analysis with adjustments for age and gender

*p* < 0.05 indicates statistical significance

Bold indicates that the values have statistical significance

In the stratification of age, *CYP3A4* SNPs rs3735451 and rs4646440 were associated with the susceptibility to IS at age > 61 years (Table 5). For rs3735451, the C allele carriers had a decreased risk of IS (OR = 0.63, 95% CI: 0.41–0.96, *p* = 0.031 for CT vs. TT genotypes; OR = 0.65, 95% CI: 0.43–0.97, *p* = 0.036 for CT-CC vs. TT genotypes) after adjusting for age and gender. For rs4646440, we found that the A allele was significantly associated with a reduced risk of IS (GA vs.GG, OR = 0.57, 95% CI: 0.37–0.88, *p* = 0.012; and GA-AA vs.GG OR = 0.60, 95% CI: 0.40–0.91, *p* = 0.017). Among the population under the age of 61, we found that *CYP3A4* rs4646437, *CYP11A1* rs12912592 and rs28681535 were associated with IS risk. *CYP3A4* rs4646437 and *CYP11A1* rs28681535 polymorphisms were significantly associated with decreased risk for IS (rs4646437, OR = 0.59, 95% CI: 0.37–0.95, *p* = 0.029; and rs28681535, OR = 0.66, 95% CI: 0.45–0.98, *p* = 0.038). Additionally, the carriers of the T allele at *CYP11A1* rs12912592 appeared to have a higher risk of IS (T vs G, OR = 1.64, 95% CI: 1.04–2.61, *p* = 0.043; GT vs GG, OR = 1.84, 95% CI: 1.11–3.05, *p* = 0.017 and GT-TT vs GG, OR = 1.89, 95% CI: 1.15–3.12, *p* = 0.013).

**Table 5:** Relationships between *CYP3A4* and *CYP11A1* polymorphism and ischemic stroke risk according to the stratification by age

SNP ID	Allele/Genotype	> 61				≤61			
		Case	Control	OR (95%CI)	*p*	Case	Control	OR (95%CI)	*p*
CYP3A4 rs3735451	T	403	287	1.00	0.063	302	399	1.00	0.217
	C	145	135	0.76 (0.58–1.01)		104	165	0.83 (0.62–1.11)	
	TT	148	92	1.00		108	136	1.00	
	CT	107	103	**0.63 (0.41–0.96)**	**0.031**	86	127	0.85 (0.58–1.24)	0.388
	CC	19	16	0.77 (0.35–1.70)	0.514	9	19	0.63 (0.27–1.47)	0.290
	CT-CC	126	119	**0.65 (0.43–0.97)**	**0.036**	95	146	0.82 (0.57–1.18)	0.288
CYP3A4 rs4646440	G	439	314	1.00	0.051	329	440	1.00	0.334
	A	109	106	0.74 (0.54–1.00)		77	122	0.84 (0.61–1.16)	
	GG	176	114	1.00		131	168	1.00	
	GA	87	86	**0.57 (0.37–0.88)**	**0.012**	67	104	0.82 (0.56–1.21)	0.321
	AA	11	10	0.86 (0.33–2.25)	0.764	5	9	0.79 (0.26–2.44)	0.684
	GA-AA	98	96	**0.60 (0.40–0.91)**	**0.017**	72	113	0.82 (0.56–1.20)	0.302
CYP3A4 rs4646437	G	486	368	1.00	0.487	360	485	1.00	0.284
	A	62	54	0.87 (0.59–1.28)		46	77	0.80 (0.55–1.19)	
	GG	214	160	1.00		163	207	1.00	
	GA	58	48	0.81 (0.49–1.32)	0.396	34	71	**0.59 (0.37–0.95)**	**0.029**
	AA	2	3	0.48 (0.06–3.65)	0.478	6	3	2.41 (0.57–10.25)	0.233
	GA-AA	60	51	0.79 (0.49–1.28)	0.335	40	74	0.67 (0.43–1.04)	0.073
CYP11A1 rs12912592	G	498	376	1.00	0.659	364	527	1.00	**0.043**
	T	50	42	0.90 (0.58–1.38)		42	37	**1.64 (1.04–2.61)**	
	GG	226	168	1.00		162	245	1.00	
	GT	46	40	0.93 (0.55–1.58)	0.798	40	37	**1.84 (1.11–3.05)**	**0.017**
	TT	2	1	0.53 (0.04–7.01)	0.629	1	0	/	/
	GT-TT	48	41	0.92 (0.55–1.54)	0.744	41	37	**1.89 (1.15–3.12)**	**0.013**
CYP11A1 rs28681535	G	298	237	1.00	0.603	246	309	1.00	0.076
	T	250	185	1.08 (0.83–1.39)		160	255	0.79 (0.61–1.02)	
	GG	75	67	1.00		80	87	1.00	
	GT	148	103	1.31 (0.82–2.09)	0.251	86	135	0.69 (0.46–1.05)	0.081
	TT	51	41	1.10 (0.61–1.99)	0.743	37	60	0.60 (0.36–1.02)	0.061
	GT-TT	199	144	1.25 (0.81–1.95)	0.318	123	195	**0.66 (0.45–0.98)**	**0.038**

SNP, single nucleotide polymorphism; OR, odds ratio; 95% CI, 95% confidence interval

*p* values were calculated by logistic regression analysis with adjustments for age and gender

*p* < 0.05 indicates statistical significance

Bold indicates that the values have statistical significance

**Fig. 1:**
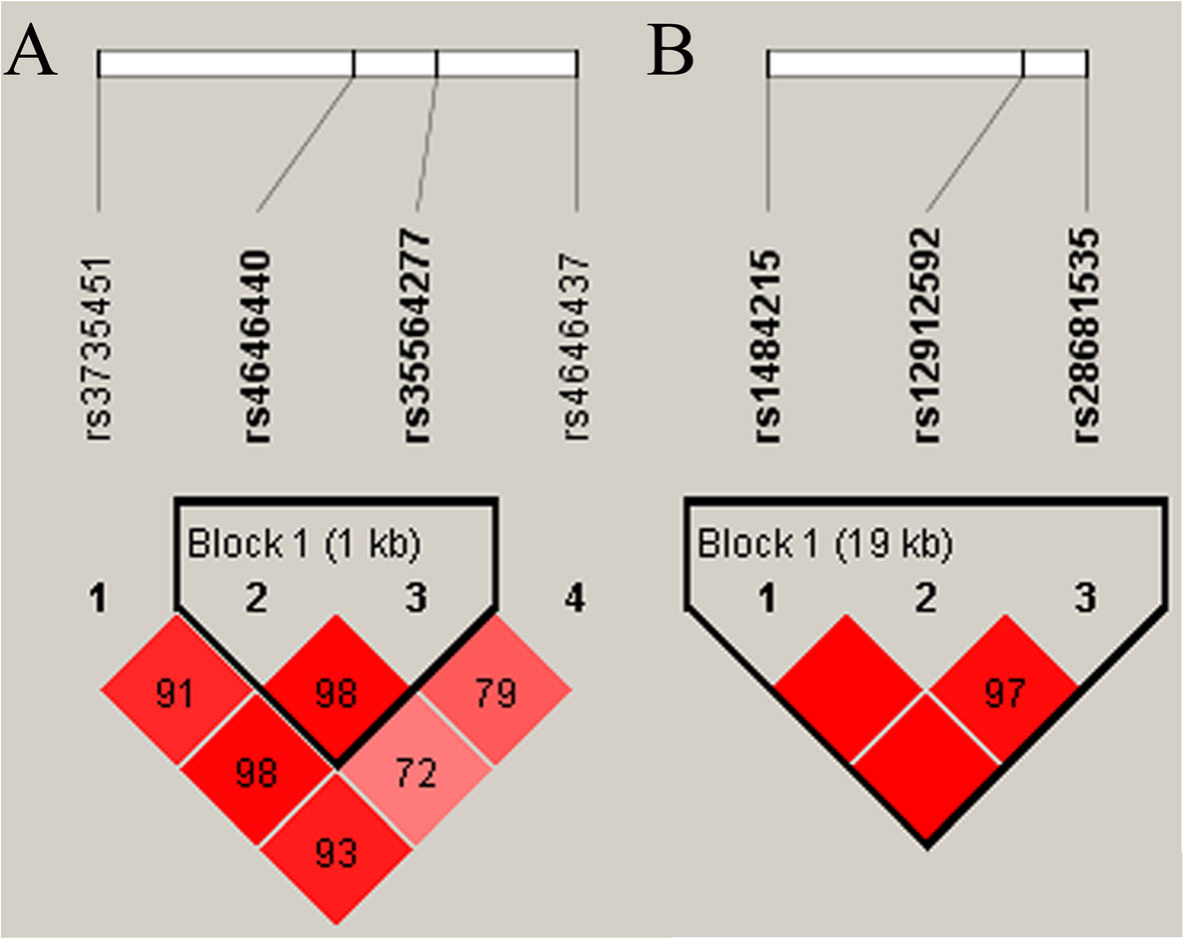
Haplotype block map for SNPs in *CYP3A4* (**a**) and *CYP11A1* (**b**) gene. Numbers in squares are D’ values in Fig. 1

We next performed haplotype analyses, and the results showed that *CYP3A4* rs4646440 was in strong linkage disequilibrium (LD) with rs35564277. Additionally, three *CYP11A1* SNPs (rs1484215, rs12912592, and rs28681535) were in strong LD, as shown in Fig. 1. However, no association of the haplotypes in *CYP3A4* and *CYP11A1* was found (Table 6).

**Table 6:** Haplotype frequencies and their associations with ischemic stroke risk

Gene	SNP	Haplotype	Frequency		Crude analysis		Adjusted by age and gender	
			Case	Control	OR (95% CI)	*p*	OR (95% CI)	*p*
CYP3A4	rs4646440|rs35564277	GT	0.805	0.768	1		1	
	rs4646440|rs35564277	AT	0.138	0.159	0.82 (0.63–1.06)	0.130	0.78 (0.60–1.02)	0.070
	rs4646440|rs35564277	AC	0.058	0.072	0.79 (0.55–1.12)	0.190	0.75 (0.52–1.08)	0.120
CYP11A1	rs1484215|rs12912592|rs28681535	CGT	0.430	0.446	1		1	
	rs1484215|rs12912592|rs28681535	CGG	0.294	0.290	1.07 (0.87–1.31)	0.550	1.06 (0.85–1.31)	0.600
	rs1484215|rs12912592|rs28681535	TGG	0.180	0.184	1.01 (0.79–1.29)	0.940	1.06 (0.82–1.37)	0.670
	rs1484215|rs12912592|rs28681535	CTG	0.096	0.080	1.29 (0.92–1.81)	0.130	1.24 (0.87–1.75)	0.230

*CYP3A4* block comprises the two closely linked SNPs rs4646440 and rs35564277. *CYP11A14* block comprises the three closely linked SNPs rs1484215, rs12912592, and rs28681535

*OR* odds ratio, *95% CI* 95% confidence interval

*p* values were calculated using logistic regression analysis with and without adjustment by gender and age

Furthermore, we also assessed the association of the selected SNPs and clinical variables in patients (Table 7). Significant association was observed between the genotypes of the *CYP3A4* SNPs rs3735451 and rs4646440 and the levels of total protein (*p* = 0.021 and *p* = 0.043, respectively). A significant association of *CYP11A1* rs12912592 polymorphism with total bilirubin was identified (*p* = 0.025). Besides, the TT genotype of *CYP11A1* rs28681535 was higher high-density lipoprotein cholesterol level than GT genotype and GG genotype (*p* = 0.027). However, there was no difference in the remaining lipid parameters among the genotypes of the selected SNPs (*p* > 0.05 for all).

**Table 7:** Comparisons of clinical characteristics among patients with different genotypes of selected SNPs

Characteristics	CYP3A4 rs3735451				CYP3A4 rs4646440			
	TT	TC	CC	*p*	AA	AG	GG	*p*
TP (g/L)	66.16 ± 6.02	64.65 ± 5.34	66.79 ± 6.09	**0.021**	66.98 ± 6.96	64.60 ± 5.33	66.02 ± 5.92	**0.043**
Serum uric acid (μmol/L)	290.64 ± 97.14	279.93 ± 93.17	260.83 ± 72.42	0.247	266.33 ± 61.31	280.39 ± 94.51	287.64 ± 95.95	0.583
Blood glucose (mmol/L)	6.36 ± 2.05	6.22 ± 2.33	6.77 ± 3.19	0.484	6.75 ± 2.92	6.35 ± 2.59	6.29 ± 2.02	0.734
TB (μmol/L)	13.78 ± 6.99	13.23 ± 5.32	14.95 ± 9.03	0.422	16.56 ± 11.62	13.36 ± 5.23	13.62 ± 6.75	0.237
TG (mmol/L)	1.55 ± 0.88	1.65 ± 1.24	1.53 ± 1.22	0.596	1.79 ± 1.43	1.60 ± 1.02	1.57 ± 1.06	0.771
Hemoglobin (g/L)	137.07 ± 21.57	137.51 ± 22.94	130.46 ± 31.22	0.395	133.57 ± 15.59	137.31 ± 25.98	136.82 ± 21.38	0.842
TC (mmol/L)	3.93 ± 0.99	3.89 ± 1.05	3.65 ± 1.19	0.464	3.92 ± 1.13	3.76 ± 1.10	3.96 ± 0.98	0.200
HDL-C (mmol/L)	1.10 ± 0.24	1.09 ± 0.26	1.03 ± 0.37	0.494	1.01 ± 0.31	1.07 ± 0.27	1.11 ± 0.25	0.203
LDL-C (mmol/L)	1.82 ± 0.56	1.82 ± 0.62	1.66 ± 0.56	0.446	1.80 ± 0.55	1.76 ± 0.65	1.84 ± 0.55	0.375
Characteristics	CYP11A1 rs12912592				CYP11A1 rs28681535			
	TT	GT	GG	*p*	TT	GT	GG	*p*
TP (g/L)	65.50 ± 0.56	65.32 ± 6.02	65.64 ± 5.78	0.910	65.72 ± 5.75	65.62 ± 5.82	65.46 ± 5.83	0.946
Serum uric acid (μmol/L)	245.50 ± 62.93	290.63 ± 99.32	284.76 ± 92.30	0.744	2810 ± 87.44	284.04 ± 93.26	290.48 ± 96.95	0.749
Blood glucose (mmol/L)	4.93 ± 0.21	6.57 ± 2.34	6.27 ± 2.23	0.397	6.85 ± 2.92	6.26 ± 2.12	6.11 ± 1.96	0.064
TB (μmol/L)	9.33 ± 2.82	12.02 ± 4.58	14.05 ± 6.84	**0.025**	13.71 ± 6.91	14.23 ± 7.35	12.75 ± 4.68	0.117
TG (mmol/L)	0.76 ± 0.39	1.65 ± 1.28	1.57 ± 0.99	0.342	1.33 ± 0.53	1.63 ± 1.05	1.63 ± 1.22	0.093
Hemoglobin (g/L)	130.67 ± 4.04	136.46 ± 27.08	136.94 ± 21.84	0.885	137.54 + 15.96	136.85 ± 24.84	136.31 ± 22.92	0.932
TC (mmol/L)	3.64 ± 0.33	3.97 ± 1.00	3.89 ± 1.02	0.774	3.96 ± 0.84	3.95 ± 1.10	3.81 ± 0.97	0.433
HDL-C (mmol/L)	1.21 ± 0.35	1.07 ± 0.24	1.09 ± 0.26	0.579	1.16 ± 0.27	1.08 ± 0.25	1.06 ± 0.24	**0.027**
LDL-C (mmol/L)	1.86 ± 0.20	1.84 ± 0.56	1.81 ± 0.59	0.871	1.88 ± 0.52	1.83 ± 0.62	1.75 ± 0.56	0.311

*SNP* single nucleotide polymorphism, *TP* total protein, *TB* total bilirubin, *TC* total cholesterol, *TG* triglyceride, *HDL-C* high-density lipoprotein cholesterol, *LDL-C* low-density lipoprotein cholesterol

*p* < 0.05 indicates statistical significance

Bold indicates that the values have statistical significance

## Discussion

The aim of this investigation was to discover whether there was an association between the *CYP3A4* and *CYP11A1* polymorphisms and IS risk in Chinese population. In this study, we found that C allele and CT genotype of rs3735451 and GA genotype of rs4646440 in *CYP3A4* were significantly associated with a reduced risk of IS in the overall. We further demonstrated that *CYP3A4* rs4646440 was associated with a decreased risk of IS, whereas *CYP11A1* rs12912592 was associated with a higher risk of IS in males. In addition, our study found that *CYP3A4* rs3735451 and rs4646440 possibly contributed to the susceptibility to IS at age > 61 years, and rs4646437 in *CYP3A4* and rs12912592 and rs28681535 in *CYP11A1* were associated with the risk of IS at age ≤ 61 years. Besides, the TT genotype of *CYP11A1* rs28681535 was higher high-density lipoprotein cholesterol level than GT genotype and GG genotype (*p* = 0.027). To the best of our knowledge, this is the first study to demonstrate the association of these polymorphisms in *CYP3A4* and *CYP11A1* with IS risk in Chinese population.

CYP genes encode monooxygenases responsible for arachidonic acid metabolism, which is involved in cardiovascular diseases and stroke [17]. Numerous studies have suggested an association between genetic variants of CYP pathway genes and the risk of IS [18]. *CYP3A4* gene encodes an enzyme, which involved in drug metabolism and synthesis of cholesterol, steroids and other lipids, and mediated the production of arachidonic acid metabolites [19, 20]. *CYP11A* gene, a member of CYP genes, encodes a cholesterol side chain cleavage enzyme (cytochrome P450 cholesterol side-chain cleavage, P450scc) that plays a major role in the control of steroidogenesis, by mediating the conversion of cholesterol to pregnenolone [21]. Dyslipidemia such as low concentration of high-density lipoprotein cholesterol (HDL-C), high levels of low-density lipoprotein cholesterol (LDL-C), total cholesterol (TC) was one of the most important risk factors of IS [22]. These lines of evidence have led us to formulate the hypothesis that *CYP3A4* and *CYP11A1* could be of pathogenic importance in IS.

Variations in the *CYP3A4* or *CYP11A1* genes may influence the gene expression, which might associate with the occurrence and progression of disease. In this study, we found that *CYP3A4* (rs3735451, rs4646440 and rs4646437) and *CYP11A1* (rs12912592 and rs28681535) polymorphisms were significantly associated with the risk of IS. These polymorphisms are located in the intron region. Chen CH er al. reported that rs4646440 was associated with higher CYP3A4 enzyme activities [23]. Reportedly, rs4646437 influences the protein expression and enzymatic activity of hepatic CYP3A4 [24]. The functional mechanism of other variants have not been reported in previous studies. The functional mechanism of these variants have not been reported in previous studies. Based on HaploReg database, we found these polymorphisms might be associated with the regulation of promoter/enhancer histone, DNAse, proteins binding and changed motifs and/or selected eQTL hits. Several studies provided increasing evidence to support that intronic SNPs confer susceptibilities by affecting gene expression [25–27]. Therefore, we hypothesized that *CYP3A4* or *CYP11A1* polymorphisms may affect the expression of their genes to contribute to the risk of IS. However, further study is necessary to confirm this hypothesis.

Stroke is a sex-specific disease and the prevalence of stroke in women is lower than that in men [28, 29]. Stratified by gender, we noticed that *CYP3A4* rs4646440 and *CYP11A1* rs12912592 polymorphism affected IS risk in males but not in females, which indicate that this risk association presented sex difference and emphasize the importance of considering heterogeneity in genetic and stroke association studies. In addition, stroke is a late-onset disease and the rate is higher in older people [30, 31]. Our study found that *CYP3A4* rs3735451 and rs4646440 possibly contributed to the susceptibility to IS at age > 61 years, and *CYP3A4* rs4646437 and *CYP11A1* rs12912592 and rs28681535 were associated with the risk of IS at age ≤ 61 years. These suggested the interactions between *CYP3A4* and *CYP11A1* polymorphisms and some environmental exposures (such as males, elder) contributed to the risk of IS.

Inevitably, our current study has some limitations to be considered. First, due to all participants were all enrolled in the same hospital, the inherent selecting bias and information bias could not be completely excluded for the group of patients with IS. Second, data deficiencies of some exposure factors such as obesity, smoking, and alcohol limited our ability to evaluate gene–environment interaction. Finally, explicit mechanisms of *CYP3A4* and *CYP11A1* polymorphism on development of IS are still bewildered and further research is needed. Despite the limitations mentioned above, the results of our present study provided scientific evidence of *CYP3A4* and *CYP11A1* gene with IS for the future studies.

## Conclusions

To sum up, our study provided evidence that variants of *CYP3A4* and *CYP11A1* gene had a significant effect on the risk of IS in the Chinese Han population, which has not previously been reported. Our study may provide clues for the evaluation of individual susceptibility to IS and increase the understanding of the possible effect of *CYP3A4* and *CYP11A1* gene on the development of IS. However, the replication of this research in different populations and additional functional analysis is required to completely elucidate the roles by which *CYP3A4* and *CYP11A1* polymorphisms predispose for IS.

## Supplementary information

**Additional file 1:Table S1.** Primers sequence of PCR and UEP used in this study. **Table S2**. Relationships between *CYP3A4* polymorphism and Ischemic stroke risk.

## Data Availability

The datasets used and/or analysed during the current study are available from the corresponding author on reasonable request.

## Abbreviations

AA: Arachidonic acid
CI: Confidence intervals
CYPs: Cytochrome P450s
EETs: Epoxyeicosatrienoic acids
HDL-C: High-density lipoprotein cholesterol
HWE: Hardy–weinberg equilibrium
IS: Ischemic stroke
LD: Linkage disequilibrium
LDL-C: Low-density lipoprotein cholesterol
MAFs: Minor allele frequencies
OR: Odds ratio
SNP: Single-nucleotide polymorphisms
TC: Total cholesterol
