# Supplementary material for: CYP3A4 and CYP11A1 variants are risk factors for ischemic stroke: a case control study
Source: BMC Neurol. 2020 Mar 4;20:77. doi: 10.1186/s12883-020-1628-4 (PMC7055027; doi:10.1186/s12883-020-1628-4)
Supplement: Supplementary file 1 — Additional file 1:Table S1. Primers sequence of PCR and UEP used in this study. Table S2. Relationships between CYP3A4 polymorphism and Ischemic stroke risk. [file 12883_2020_1628_MOESM1_ESM.docx]

Additional file 1

**Table S1. Primers sequence of PCR and UEP used in this study**

| **SNP** | **First Primer(5'-3')** | **Second Primer (5'-3')** | **UEP SEQ (5'-3')** |
| --- | --- | --- | --- |
| rs3735451 | ACGTTGGATGCAAAGTGAGTGAGACACTCC | ACGTTGGATGTACTGCATTTTTTTTGCCC | ccccTTTGCCCATTACTCCAT |
| rs4646440 | ACGTTGGATGATGCTAAGGATTTCAGTCCC | ACGTTGGATGCCAACTATGATGTGTGGAGG | cccgcTGTGTGGAGGAGTTATGAAGT |
| rs35564277 | ACGTTGGATGGGCCCAACTTGTAATCATAG | ACGTTGGATGTGGACAAAAAGCTAGATGAG | CAAAAAGCTAGATGAGTGGTAA |
| rs4646437 | ACGTTGGATGCTTCAAAAGATGCACAAGGG | ACGTTGGATGAGGGCAGGTCTATGCATAAG | ctgaAGGTCTATGCATAAGGAGCACC |
| rs1484215 | ACGTTGGATGTAGCAGGGCCTCTAGGTGA | ACGTTGGATGTGAAGGCTGGCAGAGCAATT | ccttgTTAGGGAGGCAGGAAAATGA |
| rs12912592 | ACGTTGGATGACTCCCAGGTTCAAGCAATC | ACGTTGGATGAAAATTGGCTGGGTGTGGTC | ggttTGGTCACGTGCACCTGTAATC |
| rs28681535 | ACGTTGGATGTACAGGTAAGCCTGGCAGAG | ACGTTGGATGAACCAGAGAACAGCCTGTTG | gacgACAGCAGGGCTACCCAG |

SNP: Single nucleotide polymorphism; UEP: Unextended mini sequencing primer

Table S2. Relationships between *CYP3A4* polymorphism and **Ischemic stroke** risk

| **Gene SNP ID** | **Model** | **Genotype** | **Case** | **Control** | **Adjusted by age and gender** | |
| --- | --- | --- | --- | --- | --- | --- |
|  |  |  |  |  | **OR (95%CI)** | ***p*** |
| CYP3A4  rs35564277 | Allele | T | 898 | 913 | 1.00 | 0.234 |
|  |  | C | 56 | 71 | 0.80 (0.56-1.15) |  |
|  | Genotype | TT | 426 | 425 | 1.00 |  |
|  |  | CT | 46 | 63 | 0.69 (0.46-1.05) | 0.083 |
|  |  | CC | 5 | 4 | 1.43 (0.38-5.43) | 0.601 |
|  | Dominant | TT | 426 | 425 | 1.00 | 0.129 |
|  |  | CT-CC | 51 | 67 | 0.73 (0.49-1.09) |  |
|  | Recessive | TT-CT | 472 | 488 | 1.00 | 0.560 |
|  |  | CC | 5 | 4 | 1.49 (0.39-5.65) |  |
|  | Log-additive | --- | --- | --- | 0.80 (0.56-1.15) | 0.229 |
| CYP3A4  rs4646437 | Allele | G | 846 | 853 | 1.00 | 0.190 |
|  |  | A | 108 | 131 | 0.83 (0.63-1.09) |  |
|  | Genotype | GG | 377 | 367 | 1.00 |  |
|  |  | GA | 92 | 119 | 0.74 (0.54-1.01) | 0.059 |
|  |  | AA | 8 | 6 | 1.57 (0.52-4.72) | 0.426 |
|  | Dominant | GG | 377 | 367 | 1.00 | 0.100 |
|  |  | GA-AA | 100 | 125 | 0.77 (0.57-1.05) |  |
|  | Recessive | GG-GA | 469 | 486 | 1.00 | 0.359 |
|  |  | AA | 8 | 6 | 1.67 (0.56-5.04) |  |
|  | Log-additive | --- | --- | --- | 0.83 (0.63-1.11) | 0.207 |
| CYP11A1  rs1484215 | Allele | C | 782 | 808 | 1.00 | 0.860 |
|  |  | T | 172 | 182 | 0.98 (0.78-1.23) |  |
|  | Genotype | CC | 319 | 329 | 1.00 |  |
|  |  | CT | 144 | 150 | 1.04 (0.78-1.37) | 0.813 |
|  |  | TT | 14 | 16 | 0.96 (0.45-2.05) | 0.921 |
|  | Dominant | CC | 319 | 329 | 1.00 | 0.844 |
|  |  | CT-TT | 158 | 166 | 1.03 (0.78-1.35) |  |
|  | Recessive | CC-CT | 163 | 479 | 1.00 | 0.898 |
|  |  | TT | 14 | 16 | 0.95 (0.45-2.01) |  |
|  | Log-additive | --- | --- | --- | 1.02 (0.80-1.29) | 0.896 |
| CYP11A1  rs12912592 | Allele | G | 862 | 903 | 1.00 | 0.230 |
|  |  | T | 92 | 79 | 1.22 (0.89-1.67) |  |
|  | Genotype | GG | 388 | 413 | 1.00 |  |
|  |  | GT | 86 | 77 | 1.17 (0.83-1.66) | 0.369 |
|  |  | TT | 3 | 1 | 2.09 (0.21-21.12) | 0.531 |
|  | Dominant | GG | 388 | 413 | 1.00 | 0.330 |
|  |  | GT-TT | 89 | 78 | 1.19 (0.84-1.67) |  |
|  | Recessive | GG-GT | 447 | 490 | 1.00 | 0.547 |
|  |  | TT | 3 | 1 | 2.04 (0.20-20.55) |  |
|  | Log-additive | --- | --- | --- | 1.19 (0.86-1.66) | 0.299 |
| CYP11A1  rs28681535 | Allele | G | 544 | 546 | 1.00 | 0.492 |
|  |  | T | 410 | 440 | 0.94 (0.78-1.12) |  |
|  | Genotype | GG | 155 | 154 | 1.00 |  |
|  |  | GT | 234 | 238 | 0.92 (0.68-1.23) | 0.569 |
|  |  | TT | 88 | 101 | 0.86 (0.59-1.25) | 0.425 |
|  | Dominant | GG | 155 | 154 | 1.00 | 0.461 |
|  |  | GT-TT | 322 | 339 | 0.90 (0.68-1.19) |  |
|  | Recessive | GG-GT | 389 | 392 | 1.00 | 0.549 |
|  |  | TT | 88 | 101 | 0.90 (0.65-1.26) |  |
|  | Log-additive | --- | --- | --- | 0.93 (0.77-1.11) | 0.411 |

SNP, single nucleotide polymorphism; OR, odds ratio; 95% CI, 95% confidence interval.

*p* values were calculated by logistic regression analysis with adjustments for age and gender.

*p* < 0.05 means the data is statistically significant.
